# Adaptive control ankle robotics training durably improves gait biomechanics in chronic hemiparetic stroke and footdrop

**DOI:** 10.1186/s12984-025-01834-2

**Published:** 2025-12-29

**Authors:** Anindo Roy, Bradley Hennessie, Charlene Hafer-Macko, Larry W. Forrester, Kelly Westlake, Richard F. Macko

**Affiliations:** 1 NextStep Robotics, Inc., 800 West Baltimore Street, Baltimore, MD 21201 USA; 2 Department of Mechanical Engineering and Maryland Applied Graduate Engineering (MAGE), University of Maryland, College Park, MD USA; 3 Department of Physical Therapy & Rehabilitation Science, University of Maryland, Baltimore, MD USA; 4 University of Maryland Older Americans Independence Center, Baltimore, MD USA

**Keywords:** Stroke, Hemiparetic gait, Foot drop, Robotics, Exoskeleton, Locomotor training, Task-specific training, Biomechanical adaptation

## Abstract

**Background:**

Robotics has emerged as a promising avenue for gait rehabilitation after stroke. We developed a wearable ankle exoskeleton (AMBLE) for dorsiflexion assist-as-needed training with adaptive control timing to individualize assistance across gait cycle sub-events. This single-armed, non-controlled study investigates effects of 9 weeks x 2 sessions/week robotics training on walking function in persons with chronic stroke and foot drop, and durability 2 months after training ends.

**Findings:**

Subjects included *N* = 24 participants (12 male, 12 female) age 57 ± 13 years with mean 10 ± 9 years since stroke. All baseline and post-training outcomes included optical motion capture for 3-D gait biomechanics and were conducted during unassisted (no robot) over-ground walking conditions. AMBLE training improved select gait biomechanics outcomes including maximum toe clearance (mm, pre- 69±28 versus post- 79±30, *p* < 0.01), ankle peak dorsiflexion angular velocity (°/s, 35±32 versus 47±40, *p* < 0.01), heel-first foot strikes (%steps, 31±41 versus 44±43, *p* < 0.01), and paretic step length (cm, 37±16 versus 40±14, *p* < 0.01. Functional outcomes that improved with training included 10-meter self-selected (m/s, 0.66±0.24 versus 0.70±0.23, *p* < 0.01) and fastest comfortable velocities (m/s, 0.80±0.31 versus 0.86±0.30, *p* < 0.01), Dynamic Gait Index (points, 14±5 versus 17±3, *p* < 0.01), 6-minute walk distance (m, 252±106 versus 280±109, *p* < 0.01), and Stroke Impact Scale-Mobility (0-100, 280±109 versus 88±10, *p* < 0.01); all achieving minimal clinically important differences, except walking velocities. Durability testing 2 months after cessation of robotics training showed retention of most biomechanical improvements, including maximum toe clearance (mm, 78.4±26.1, *p* < 0.01), dorsiflexion angular velocity (°/s, 42.5±37.5, *p* < 0.01), and heel-first foot strikes (%steps, 46±43, *p* < 0.01), and most functional outcomes. Notably, durability testing revealed emergence of two new kinematic improvements: increased knee flexion (deg, 33.1±16.5 versus 36.9±17.7, *p* < 0.01) and hip flexion (deg, 36.8±9.5 versus 39.6±9.2, *p* < 0.05), while hip abduction and hip hike do not change.

**Conclusions:**

Nine hours of AMBLE ankle robotics training across 9 weeks durably improves gait biomechanics and functional mobility for persons with chronic stroke and foot drop, even decades post-stroke. Further studies are needed to investigate precision adaptive control robotics for stroke and other mobility disability conditions.

*Trial registration*: Clinical trial identifier: NCT04594837.

## Background

Stroke is a leading cause of long-term disability, with hemiparetic gait (HPG) being one of the most persistent and debilitating consequences [1]. The defining elements of HPG are reduced cadence, step length asymmetry, prolonged swing time on the paretic side, and increased stance time on the non-paretic side [2, 3]. These gait abnormalities often coexist with impaired ankle motor control, sub-optimal foot landing, and reduced balance; all of which compromise function and safety [4, 5]. Among these deficits, foot drop, affecting approximately 30% of stroke survivors, is on the more severe end of the spectrum of chronic HPG deficits. It results in inefficient gait mechanics and heightened fall risk due to inadequate toe clearance during swing phase [6]. However, foot drop rarely occurs in isolation; it interacts with other HPG elements and compensatory strategies involving knee and hip motor control to influence the overall biomechanical characteristics of gait, functional mobility, and fall risk. Despite its clinical relevance, foot drop remains a challenging therapeutic target, it is often addressed with assistive devices rather than interventions that promote neuroplasticity.

Robotics assisted rehabilitation has emerged as a promising approach to address severe HPG manifestations like foot drop. Advanced engineering control systems, particularly impedance control, which provide assistance-as-needed while allowing natural movement (back-drivability), have emerged as a leading model for human robotics cooperative learning. Impedance control has proven effective for upper extremity recovery after stroke, influencing national care guidelines [7]. However, translating this success to locomotor rehabilitation has proven more elusive due to the need for closed-loop sensorimotor learning and the heterogeneity of gait deficits. Early robotic approaches leveraging central pattern generators that enforced symmetry or lacked volitional engagement proved limited in achieving meaningful locomotor learning outcomes, sometimes even inferior to conventional rehabilitation. This led to a shift towards end-effector or modular approaches to enable non-holonomic approaches to account for deficit heterogeneity, and actuation profiles to address sensorimotor neuroplasticity across specific sub-segments of the gait cycle.

Our prior randomized studies demonstrated the efficacy of task-specific treadmill-based training using a wearable impedance control exoskeleton (“Anklebot” [8]), to improve over-ground gait [9, 10]. However, these studies were limited by the laboratory-grade predecessor device’s heavy weight and tethering cables, restricting its usage to controlled environments [8].

There is a gap in understanding whether robotics training, particularly those with advanced control systems such as adaptive control, can mediate durable biomechanical adaptations to improve the safety and efficiency of HPG after stroke, and how long after an index disabling stroke that such robotics mediated sensorimotor learning may be clinically impactful [8–11]. Most lower extremity robotics assisted rehabilitation had focused on the immediate pre-post training changes in gait, and not the adaptive biomechanical patterns that may evolve across the months after all robotics training had ended, which ultimately will define added clinical value.

To overcome these limitations, we developed AMBLE (*A*ssisted *M*obility and *B*alance for the *L*ower *E*xtremity), a lightweight (1.4 kg), wearable powered and Bluetooth operatable ankle exoskeleton with an innovative front-mounted design [12]. AMBLE employs adaptive impedance control to deliver precise dorsiflexion assistance across three sub-phases of the swing phase: dorsiflexion rise post toe-off, mid-swing hold, and controlled dorsiflexion at initial contact to mitigate foot slap [13]. Initial support is calibrated to each person’s gait and progresses toward volitional (versus robot control) according to their ongoing abilities, as indicated by the step-by-step and cumulative measure of kinematic autonomy. Unlike soft exoskeletons that struggle with force generation and alignment or rigid systems that impose unnatural movement patterns and require complex setups, AMBLE offers a streamlined solution prioritizing functional recovery through task-specific training.

AMBLE represents a significant advancement in ankle rehabilitation through its unique non-anthropomorphic modular design and targeted assist-as-needed approach to dorsiflexion weakness. While soft exoskeletons like the Exosuit and T-FLEX rely on cable-driven or elastic materials, and rigid systems such as the MIT Anklebot, and Electro-hydraulic Ankle-Foot Orthosis (EHO) require complex body-conforming structures, AMBLE’s innovative front-mounted design eliminates these constraints [10, 14–17]. The device effectively addresses some common challenges faced by soft exoskeletons, including thermophysiological aspects of heat and moisture accumulation producing discomfort as a barrier to their adaptation [18]. Similarly, it overcomes the limitations of rigid systems that necessitate complex setup procedures involving knee braces and custom footwear [19]. AMBLE’s focused approach to dorsiflexion assistance, delivered through its streamlined design, enables precise movement control while preserving natural motion in other planes. It offers a significantly lighter alternative to conventional rigid systems, such as the Achilles exoskeleton and autonomous leg exoskeleton that require 1000–1500 g per leg plus an additional 2500–5000 g of waist-mounted components [20–22]. AMBLE successfully addresses the limitations of soft exoskeletons, such as insufficient force generation and complex control requirements due to non-linear material properties [10], while avoiding the motion restrictions and alignment challenges common to rigid systems like the MIT Anklebot [10]. Furthermore, AMBLE’s rehabilitation model emphasizes supervised therapy sessions designed to promote neuroplasticity, providing a more effective approach compared to both soft systems like T-FLEX with their complex tendon mechanisms and rigid systems that tend to impose rather than facilitate natural movement patterns [19]. This comprehensive design philosophy results in a more practical and efficient rehabilitation tool that prioritizes functional recovery over passive assistance.

This study investigates AMBLE’s ability to retrain ankle dorsiflexion and improve gait biomechanics in individuals with chronic HPG and foot drop. Over 9 weeks (2 sessions/week), participants underwent supervised training targeting dorsiflexion across specific gait sub-phases, with progress monitored through kinematic autonomy measures. Biomechanical and functional outcomes were assessed twice at baseline, post-training, and two months later to evaluate durability. We hypothesized that AMBLE will not only improve foot-drop specific outcomes, such as increased paretic ankle angles, but also induce adaptations along the kinematic chain while revealing potential compensatory strategies during the durability phase.

## Methods

*Study design*: This was a single arm study in 24 subjects with chronic stroke and foot drop. Subjects provided written informed consent using the Human Ethics and Consent to Participate Form. Salus Institutional Review Board approved the protocol and the informed consent form (Salus 22226-1U44NS111076-01; Portable Ankle Robotics). Subjects underwent screening to ensure study criteria were met, which included review of medical and neuro-imaging records, medical and neurologic exams. Baseline neurologic exam characterized deficit severity and stroke subtype based on clinical and radiographic data. Exams screened for medical and cardiopulmonary safety for walking with the AMBLE device. Eligible subjects received 9 weeks of AMBLE therapy (2 sessions per week, ~ 40 min duration per visit).

*Study Inclusion/Exclusion Criteria*: Inclusion criteria consisted of: (i) unilateral ischemic or hemorrhagic stroke, (ii) > 6 months post-stroke, (iii) age > 18 years, (iv) medical clearance, (v) residual hemiparesis of the lower extremity that included symptoms of foot-drop (see criteria below*), (vi) completed all conventional therapy, (vii) ability to ambulate at least 5 feet without an ankle foot orthosis (AFO) with their usual cane or walker, but no more than minimal contact assistance, (viii) ability to follow a 3-step command. *Dorsiflexion deficits: For the purposes of study inclusion, dorsiflexion (DF) deficits were defined based on active range of motion (AROM) and strength. Mean maximal DF AROM was measured in duplicate by trained staff using goniometry with subjects seated on plinth with knees at edge, lower legs dangling at 90º. DF deficit criterion was met when subjects were unable to reach zero degrees (foot parallel to floor, perpendicular to shank). Manual muscle testing was performed in the same seated position with inclusion criteria between grade 1/5 (trace) to 4/5 (reduced strength) in DF. Though AFO use is consistent with DF deficits, there are many factors (e.g. compensatory gait strategies) that influence this status. Exclusion criteria consisted of: (i) cardiac history of unstable angina, recent (< 3months) myocardial infarction, congestive heart failure (NYHA category II), or hemodynamically significant valvular dysfunction, (ii) hypertension that was a contraindication for routine physical therapy (> 160/100 on two assessments), (iii) medical history included recent hospitalization (< 3 months) for severe medical disease, symptomatic peripheral arterial occlusive disease, orthopedic or chronic pain conditions that significantly alter gait function, pulmonary or renal failure, or active cancer, (iv) history of non-stroke neuromuscular disorder restricting gait, (v) aphasia or cognitive functioning that confounds participation, defined as unable to follow 2-step commands or judgment of the clinician.

*Assessments and outcomes*: To establish efficacy in this chronic stroke population, we had participants serve as their own pseudo-controls by double baseline testing with two sets of outcome tests conducted one week apart. All testing was conducted at NextStep Robotics. Testing included the NIH Stroke Impact Scale (SIS version 3.0); timed 10-meter walk (gait velocity) and 6-minute walk tests (6MWT distance); Modified Dynamic Gait Index (DGI score); and gait biomechanics acquired using motion capture system (OptiTrak™) during unassisted over-ground walking. Table 1 lists the primary and secondary outcomes. The use and type of assistive device and/or ankle orthoses were recorded at each training visit. Post-testing was conducted after the 18 training sessions were completed. Durability or retention testing was conducted two months after all training had ended. Both post-testing and retention testing included the same outcome assessments that were administered during baseline. For variables of gait biomechanics, a mean of all pooled steps from at least 3 trials was utilized. Two derived variables i.e., step length ($\:{SI}_{SL}$) and stance duration symmetry ($\:{SI}_{SD}$) indices were calculated as.

**Table 1:** List of primary and secondary clinical performance outcomes

Domain	Outcome
*Functional*	**Dynamic Gait Index** (0–24)
	**6-Minute Walk Test** (m)
	**10 m Comfortable Speed** (m/s)
	**10 m Fastest Speed** (m/s)
*Ankle-Foot Control*	**Peak Swing Angle** (°)
	**Angle at Initial Contact** (°)
	Maximum Paretic Toe Clearance (mm)
	Minimum Paretic Toe Clearance (mm)
	**Paretic Heel-First Strikes** (%steps)
	Maximum Paretic DF Angular Velocity (°/s)
*Proximal Joint Control*	Paretic Hip Flexion (°)
	Paretic Hip Hike (cm)
	Paretic Hip Abduction (°)
	Paretic Knee Flexion (deg)
*Gait Temporal-Distance*	Paretic Step length (cm)
	Paretic Swing (%)
	Double Support Stance (%)

Outcomes in **bold** are the primary outcomes

1 $$\:\%{SI}_{SL}=100\times\:\left(1-\frac{{SL}_{P}}{{SL}_{NP}}\right),\:\%{SI}_{SD}=100\times\:\left(\frac{{SST}_{P}}{{SST}_{NP}}\right)$$

where $\:SL$ and $\:SST$ denoted step length and single support stance duration, respectively, and subscripts “*P*” and “*NP*” denoting the paretic and non-paretic sides, respectively.

*Trainer Eligibility & Credentialing*: The AMBLE is intended for supervised gait training by credentialed trainers in clinical settings. Trainers met one or more criteria: State-licensed physical therapists (PTs) including but not limited to board certifications in neurologic physical therapy, who were credentialed to practice at their clinic or hospital. In addition to PTs, exercise physiologists with Exercise Physiology or Exercise Science Master of Science were also authorized to use the device. Trainers underwent training on proper use of the AMBLE device in accordance with the “AMBLE Clinician Training Plan,” which included two visits. The first visit included introduction to the device, demonstration of its entire workflow, and supervised hands-on practice. The second visit consisted of a graded quiz to verify knowledge retention and clinicians performing the entire workflow without supervision. A competency exam was administered at the end with a score of 80% or higher required to be credentialed. Trainers had required times for donning and doffing the robot (don < 5 min, doff < 2 min, and emergency doff < 30 s for medical emergency situations).

**Fig. 1:**
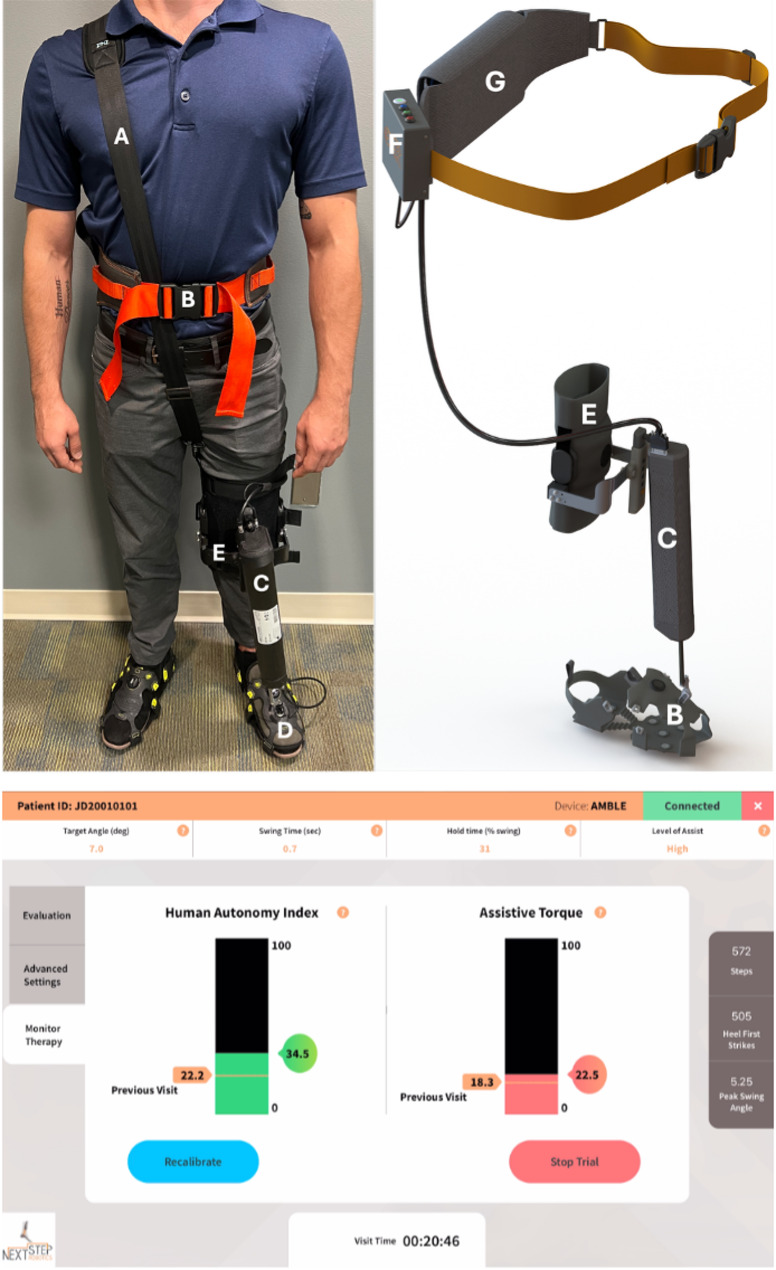
(Top) AMBLE device and its key sub-systems [12]. **A** Shoulder strap, **B** gait belt, **C** actuator unit, **D** sensor crampon, **E** knee brace, **F** control box, **G** battery (not shown). (Bottom) User app showing a snapshot of real-time outcomes and therapy inputs (top panel widgets)

*Apparatus*: AMBLE is a Class I body-worn, powered exerciser intended to restore ankle function during walking (Fig. 1) [12]. It is worn unilaterally on the affected side, provides active mechanical assistance to facilitate swing dorsiflexion, and is customized to each user’s volitional ankle strength and range of motion, to allow users to engage in treadmill or indoor over-ground walking exercises. Described below are its key features on design, actuation, control and sensing, safety, and usability.

*Kinematic design*: AMBLE is a one degree-of-freedom (DOF) wearable robot, back-drivable with low intrinsic mechanical impedance, that weighs less than 1.4 kg. It allows normal ROM in all three DOF’s of the foot relative to the shank during walking overground or on a treadmill. The AMBLE provides actuation in one of the ankle’s three DOF’s, namely dorsiflexion via a single linear actuator. AMBLE allows 25º of dorsiflexion and 35º of plantarflexion free ROM at the ankle, limits that are near the maximum range of comfortable motion for normal subjects and beyond what is required for typical gait. It has low friction (~ 6.6 N) and inertia (total of 1.4 kg at the foot) to maximize the back-drivability.

**Fig. 2:**
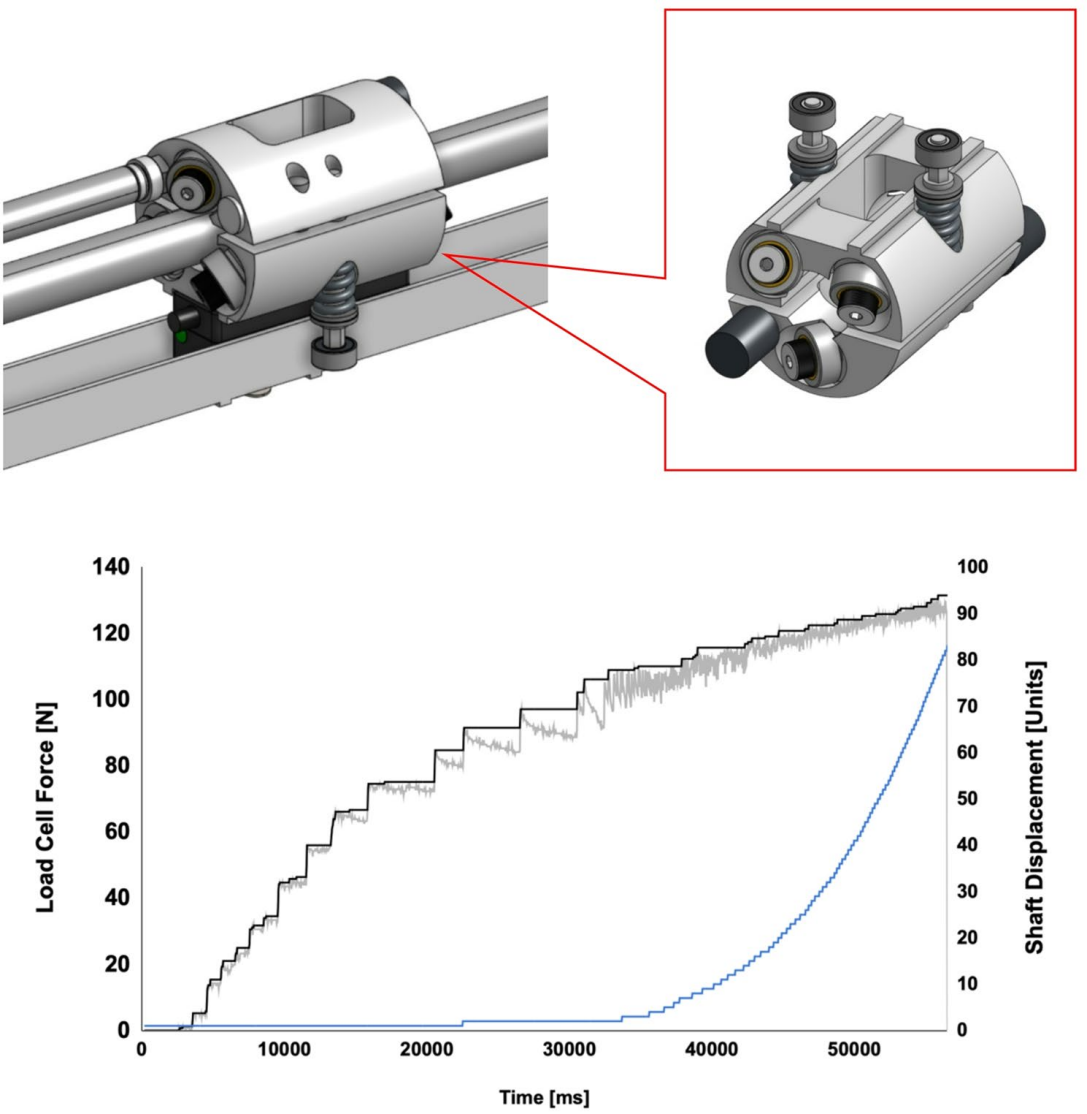
(Top) CAD Roh’Lix actuator mounted on motor driveshaft inside linear actuator. (Bottom) Load cell force and shaft displacement rotary encoder count for peak thrust verification

*Power and actuation*: The device can deliver a continuous net torque of approximately 20Nm in dorsiflexion, which is higher than required to position the foot in dorsiflexion during mid-swing. Power is supplied via high capacity, rechargeable battery (2600mAh, Inspired Energy). AMBLE is actuated by a single brushless DC motor (Maxon EC-i 40, Maxon, Switzerland), capable of generating 0.224Nm nominal and 2.1Nm stall torque, respectively, which is amplified (226 m^− 1^) and transmitted to the foot piece via a traction drive consisting of a custom, threadless linear screw actuator (NextStep Robotics, MD). The threadless linear actuator mechanism converts rotary motion into linear motion while providing adjustable torque overload protection. It’s mechanism of action utilizes six preloaded rolling element ball bearings (Fig. 2), which engage a rotating driveshaft at a pitch angle of 33.5°. As the driveshaft rotates, the bearings follow a virtual helical screw thread pattern (hence, *Ro*lling *H*el*ix*, or Roh’Lix), resulting in linear motion along the shaft. The mechanism features a nominal lead length of 25 mm/rev and a transmission ratio of 250 m^− 1^. The thrust capacity of the Roh’Lix is adjustable via thrust adjustment screws, which modify the compressive spring force that secures the two halves of the actuator chassis upon the driveshaft. If the thrust capacity is exceeded, the ball bearings slip on the smooth rotary shaft until the overload is resolved, providing an inherent safety mechanism to prevent over-torque. The actuator’s peak rated thrust capacity is 133 N (Fig. 2) as characterized by the occurrence of steady-state slip under isometric bench tests. During these tests, the thrust is measured using a miniature force sensor (Futek, LCM300, ± 50lbf) mounted in-line between the end-effector and foot mock-up. Due to the threadless interface and the large lead length of the virtual screw, the linear actuator is highly back-drivable, allowing the actuator to respond passively to the user’s intentional movement when needed.

**Fig. 3:**
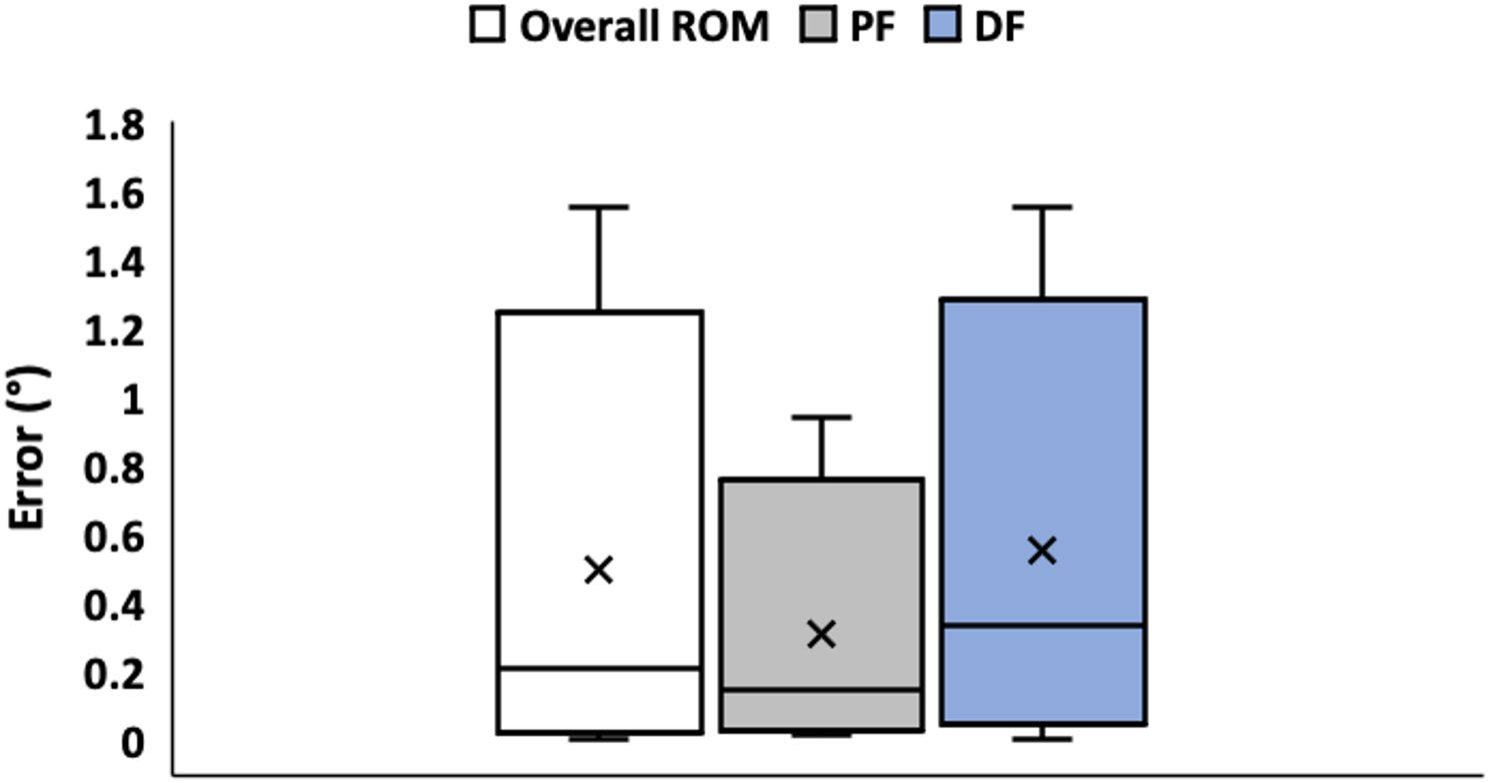
Box and whisker plot shows model-based estimation errors against motion capture measurements of ankle angles in dorsiflexion (DF) and plantar-flexion (PF)

*Motion and event sensing*: AMBLE employs a linear absolute magnetic encoder (LA11, RLS, Komenda, Slovenia) with a resolution of 3.9 μm mounted on the traction drive. The linear excursion measured by the encoder is used as feedback to the controller, to estimate ankle angle in dorsi-plantarflexion using a nonlinear model i.e.,

2 $$\:\theta\:={sin}^{-1}\left\{1-\frac{1}{2}{\left(\frac{x}{r}\right)}^{2}\right\}+{\theta\:}_{0}$$

where, $\:x$ is linear displacement (cm), $\:r$ is the distance from the point of foot attachment to the ankle joint (nominal 15 cm), and $\:\theta\:$ and $\:{\theta\:}_{0}$ are the actual and offset ankle angles (°), respectively. The kinematic model was verified using procedures described elsewhere [8]. Briefly, reflective, motion-capture markers were placed on a shank-and-foot apparatus to mimic the human leg. The device was commanded to actuate across a range of dorsiflexion (0° to 25°) and plantar-flexion (-35° to 0°) target angles. The steady-state angle readouts from the firmware were compared against values acquired using the OptiTrak™ motion capture system, as well as manual goniometry. Across the entire range of commanded angles, the overall error between the model estimates and the motion capture system was less than 1° (Fig. 3). Similarly, device torque is estimated using an empirical, linear model relating motor torque to pulse width modulation (PWM) duty cycles. Gait events are detected by an over-the-shoe crampon, which are relayed to the controller for precisely timed actuation to aid swing dorsiflexion control (see Gait Event Detection).

**Fig. 4:**
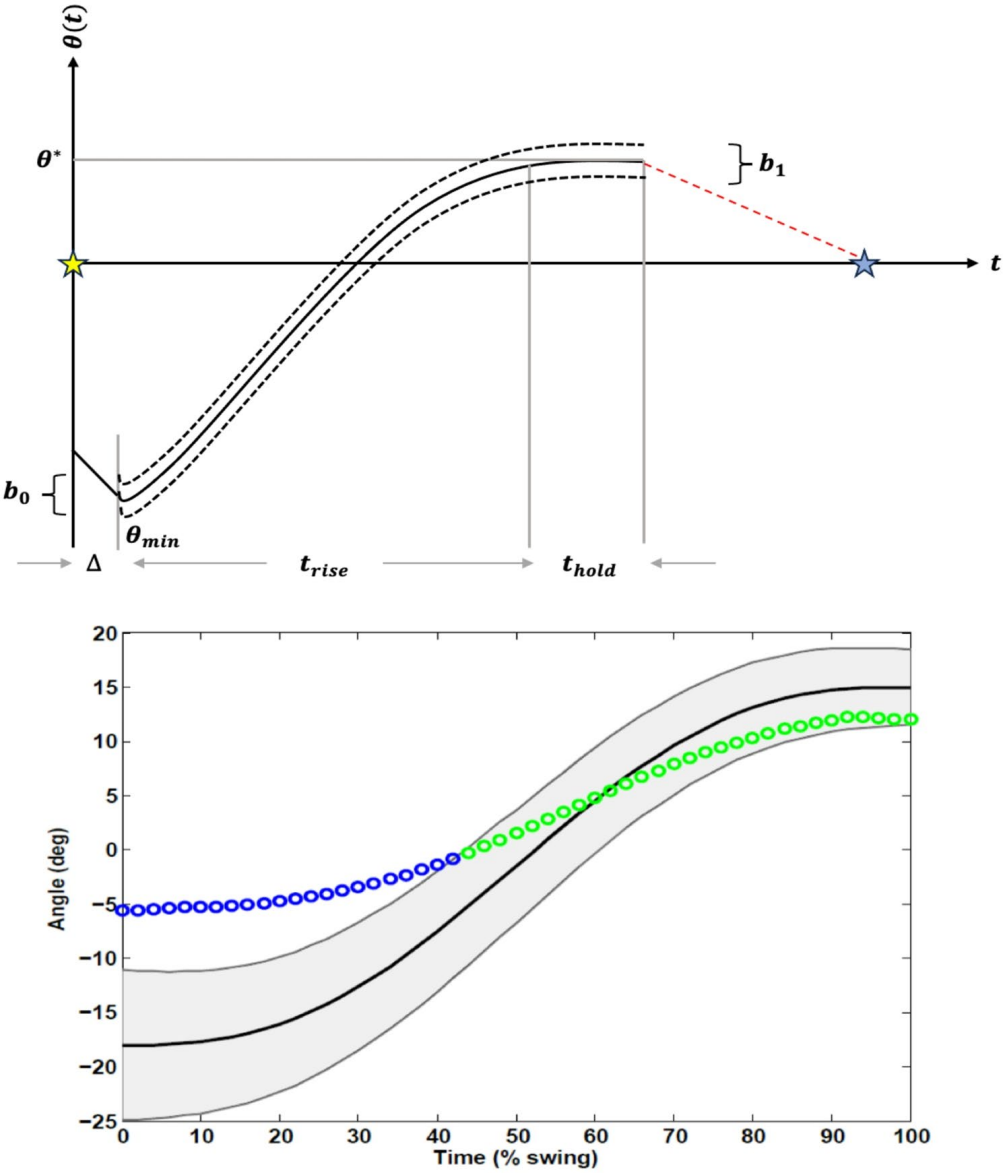
Schematic of different swing control strategies. (Top) Assist as-needed (slot) during foot rise, position control during foot hold, and damped control during foot descent. Unaided ankle angles captured during walking evaluation estimate these parameters using a linear ramp-and-hold model. (Bottom) Exemplar data from swing phase during single step: top and bottom trajectories (grey ) are created around the reference trajectory (solid **—**) to render a no-assist region (grey). Only when ankle position is either below the bottom or above the top () edges will subject receive active assistance, otherwise zero assistance is provided (green )

*Control*: The AMBLE employs multiple intra-phase control mode to address functional distinct needs with the swing phase (Fig. 4). During initial-to-mid swing, a simple impedance controller with a programmable reference position, a programmable proportional gain (approximating a controllable torsional stiffness), and a programmable derivative gain (approximating a controllable torsional damping in parallel with the stiffness) is deployed [8, 13]. During this epoch, a minimum jerk trajectory is employed as a programmable reference, around which a programmable force field is created i.e.,

3 $$\begin{aligned} \theta _{{ref}} \left( t \right) =\, & \theta _{{min,DF}} \\ & + \begin{array}{*{20}c} {\left( {\theta ^{*} - \theta _{{min,DF}} } \right)} \\ {\left\{ {10\left( {\frac{t}{{t_{{rise}} }}} \right)^{3} - 15\left( {\frac{t}{{t_{{rise}} }}} \right)^{4} + 6\left( {\frac{t}{{t_{{rise}} }}} \right)^{5} } \right\},~t_{{TO}} } \\ \end{array} \\ & + {{\Delta }} \le t < t_{{hold}} \\ \end{aligned} $$

where $\:{\theta\:}_{ref}$, $\:{\theta\:}_{min,DF}$, and $\:{\theta\:}^{*}$ are the reference trajectory, minimum angle after toe-off ($\:{t}_{TO}$), and the target dorsiflexion angle, respectively, and $\:{t}_{rise}$, $\:{t}_{hold}$ and $\:\varDelta\:$ are the rise time, hold time, and the latency following toe-off to allow gravito-inertial passive mechanics prior to foot lift (Fig. 4). Active assistance as-needed (AAN) is rendered by time-varying, upper and lower boundaries around the reference to reflect “normative” ankle movements such that no forces are generated by the device within these dynamic boundaries to promote autonomy (Fig. 4). Hence, AAN strategy acts as a point controller with respect to the boundaries but not the reference to facilitate autonomy in swing clearance. During mid-swing however, the device employs a point controller to briefly brace the ankle at peak dorsiflexion. Finally, during the late swing epoch, a damping controller may be employed to alleviate impact forces at initial contact and promote heel-first contacts. A benefit of this strategy is that the *user* rather than the *device*, dictates the timing of assistive torques to synchronize the robot’s and the user’s intents in the next step. The corresponding control laws for each epoch are as follows:

4 $$\:{\tau\:}_{c}=\left\{\begin{array}{c}{K}_{p}\left({\theta\:}_{ref}^{T}|{\theta\:}_{ref}^{B}-\theta\:\right)-{K}_{d}\dot{\theta\:},\:\varDelta\:<t\le\:\varDelta\:+{t}_{rise}\:\\\:{K}_{p}\left({\theta\:}^{*}-\theta\:\right),\:\varDelta\:+{t}_{rise}<t\le\:\varDelta\:+{t}_{rise}+{t}_{hold}\:\\\:{-K}_{d}\dot{\theta\:},\:t>\varDelta\:+{t}_{rise}+{t}_{hold}\end{array}\right.$$

where $\:{K}_{p}$ and $\:{K}_{d}$ are the proportional and derivative controller gains, $\:{\theta\:}_{ref}^{T}$ and $\:{\theta\:}_{ref}^{B}$ are the top and bottom dynamic boundaries around $\:{\theta\:}_{ref}$, and $\:\theta\:$ and $\:\dot{\theta\:}$ are the ankle angle and angular velocity, respectively. The dynamic boundaries are symmetrically created as:

5 $$ \begin{gathered} \theta _{{ref}}^{T} \left( t \right),~\theta _{{ref}}^{B} \left( t \right) = \theta _{{ref}} \left( t \right) \pm h\left( t \right)/2, \hfill \\ h\left( t \right) = b_{0} + \left( {b_{1} - b_{0} } \right)t/t_{{rise}} , \hfill \\ t_{{TO}} + {{\Delta }} \le t < t_{{hold}} \hfill \\ \end{gathered} $$

where $\:h\left(t\right)$ is the dynamic (collapsing) height of the slot, and $\:{b}_{0}$ and $\:{b}_{1}$ are the initial and final slot heights.

**Fig. 5:**
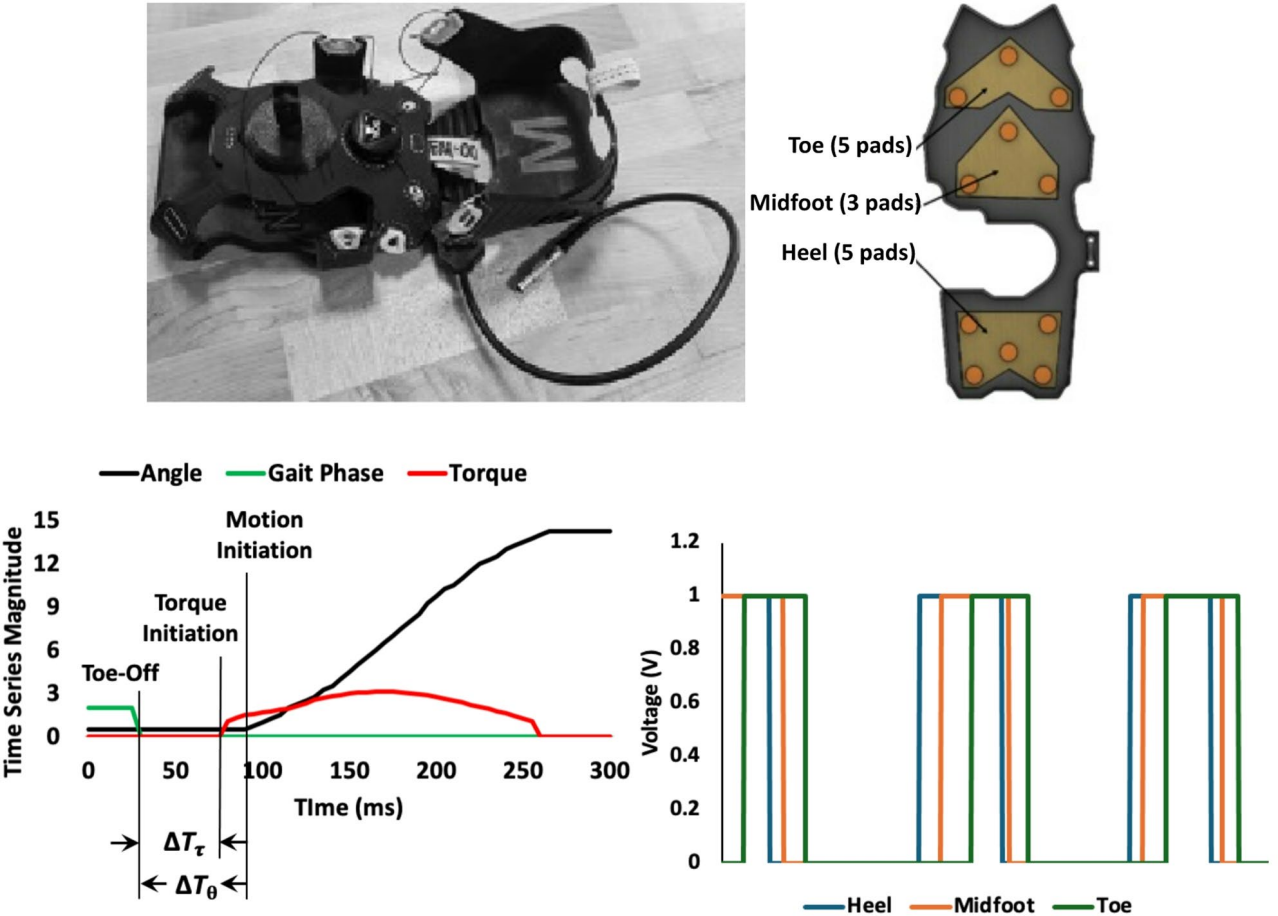
(Top) Sensor crampon and contact sensor pad layout [23]. (Bottom left) Filtered gait phase, motion, and torque data from a single step showing detection latency (Δ*T*_*τ*_, Δ*T*_*θ*_). (Bottom right) triggered logic signals from the 3 sensor regions shown for 3 steps (heel , midfoot , toe )

*Gait Event Detection*: Overlaid on Short Interval Control (SIC) and AAN strategies is precise event-based actuation that accounts for the step-to-step variability in hemiparetic gait [23]. AMBLE deploys an over-the-shoe instrumented crampon to reliably detect gait events as feedback to the controller. The crampon detects and tracks gait events via the 3 sensor regions of the sensor crampon (Fig. 5). Each crampon consists of four components: (1) two highly conductive material sheets made of brass and adhered on opposite sides of a foam sheet made of polyurethane; (2) three pads made of conductive rubber (silicon elastomer with a conductive Ni-Gr powdered filler) strategically placed in holes that represent areas implicated during foot contact, mid-stance, and toe-off; (3) a force-invoked, push-button switch that creates an electrical short, if the sensor is compressed in that given region; and (4) a rubber shell, which stabilizes the internal electrical components, protects the electronics from external environmental factors, and firmly anchors the sensor to the adjustable overshoe. The five metal sheets are adhered on opposite sides of a foam sheet consisting of holes, embedded strategically at the 3 regions, where pads of thinner, less compressible conductive rubber are placed. When the sensor is compressed, the foil sheets move towards each other until they contact the conductive rubber, creating an electrical short that is detected to determine if the sensor is compressed in that given area. The individual signals are subsequently converted to an overall logic signal (Fig. 5).

Event detection accuracy is quantified using two metrics: (1) detection latency i.e., the time between the occurrence of an event (e.g., toe-off) and the initiation of motion, as detected by the linear encoder; and (2) detection accuracy i.e., the incidence of erroneous (e.g., double) step counts (%steps). Bench verification consisted of mimicking the swing phase without end-effector load, by manually loading-unloading (step on-and-off) the crampon on the ground (simulated gait “step”). Of the 254 simulated steps, there were 253 recorded triggers (0.39% misses) with an average detection latency of ~ 63ms, the latter comparable to proprioceptive delays (50–60 ms) (Fig. 5). The incidence of multiple step counts was collected from 3 treadmill walking trials by a healthy volunteer and was < 7.3%.

*Safety*: AMBLE includes safety redundancies in multiple sub-systems and in its don/doff procedures. During don and doff, the client remains seated eliminating any potential for falls. In case of emergency, the device can be removed from the client in less than 30 s. The software continuously monitors torques, velocities, and displacements and disables the system in case preestablished limits are exceeded or in case of critical component malfunctions (e.g. linear encoder). Furthermore, the traction drive operates as a mechanical “fuse,” and it slides above preset torque values (96–116 N). For redundancy, over-torquing is also prevented by an electrical fuse (10 A) and a software threshold (7Nm). Additionally, a “stop” button on the control box is available to manually stop the device. Load distribution verification tests demonstrated that the knee brace can sustain axial loads > 200 N. Finally, AMBLE’s communication protocol includes Advanced Encryption Standard (AES) in Counter (CTR) mode using 128-bit blocks (AES-128-CTR) encryption and a sophisticated key management system to meet cybersecurity requirements.

*Usability*: The device includes a number of design features to facilitate its integration and use in clinical workflow. Excluding the time needed to determine the person’s knee brace and shoe sizes, the donning process requires < 5 min by a single clinician. Multiple sizes (small/medium/large) of the knee brace and crampons are available to accommodate individuals with different anthropometric dimensions and for optimal comfort. Further, the flexible rubber crampon is highly adjustable via a Boa dial mechanism to conform to a broad range of foot sizes (Men: 7–14, Women: 8–12). The crampon is quick to don/doff (< 10 s) and is designed to accommodate regular wear/tear (> 3000 mating cycles). Further, AMBLE’s rechargeable battery has a long continuous runtime of ~ 11 h, roughly 44 units of direct patient contact hours. AMBLE’s software app provides the ability to characterize unaided gait parameters to nominally set (see Walking Evaluation) and adjust therapy inputs step-to-step. The app also outputs step-to-step end-of-session performance outcomes for ankle control, steppage, and device use, accessible via a secure cloud.

*Reliability*: Device reliability was characterized as the type and incidence of malfunctions (%visits), which were recorded throughout the study. Clinical feasibility was quantified as the number of use case errors made by the trainers. The type and number of adverse (AEs) and serious adverse events (SAEs) were logged to determine device safety.

*Intervention*: Training sessions were conducted at NextStep Robotics by two exercise physiologists who were trained in the use of the AMBLE device. Participants received a total of 18 supervised treadmill (~ 20 min) and over-ground (~ 20 min) walking sessions while receiving assistance as-needed from the AMBLE device across a 9-week time-period. Individuals that missed three consecutive training sessions were excluded. Each visit required ~ 1 h that included: (1) pre-gait activities, which occur without the robot (don, calibration), (2) treadmill and over-ground gait with the AMBLE device in active assist mode; (3) post-gait activities, which occur without the robot (doff). Pre-training activities consisted of checking user dimensions for device sizing (knee brace, crampon, shoulder strap). Any participant whose anthropometric characteristics prevented fit (e.g., foot size outside the range of available crampon sizes) were excluded at this stage. Trainers removed any AFOs and functional electrical stimulation (FES) devices worn by participants prior to donning and recorded the time it took to put the device on the person. Additionally, on the first day of training, after fitting the robot to the user, subjects walked for 10 steps with the robot in “evaluation,” “record only” mode. Ankle trajectories from these record-only trials were used to approximate the spatial (minimum angle following toe-off and peak swing dorsiflexion) and temporal (rise and hold times) parameters to characterize a nominal reference trajectory in the software app for each participant (see Figs. 1 and 4). These parameters were set as initial therapy inputs and progressed as needed over the course of the intervention. The between-session use and type of assistive device and/or ankle or other orthoses were also recorded at each visit.

Both treadmill and over-ground walking consisted of scripted recommendations. During over-ground walking, subjects walked at their comfortable self-selected pace, completing about 20 min. Subjects were allowed to use their assistive devices (AD) for safety, if needed. Any AD used during each training visit was recorded. Treadmill training consisted of walking on an instrumented treadmill (Biodex GT3) at comfortable speed with handrail support, completing about 20 min. Subjects were informed that their total walking time (over-ground and treadmill) would be targeted at 40 min, as tolerated, but would not exceed 45 min. A Polar Heart Rate Monitor was used during the supervised walking with the maximal allowed heart rate during walking < 50% of heart rate reserve, based upon the formula of Karvonen, adjusted for any chronotropic medication usage (e.g. beta blockers), as by convention. The purpose was to assure that participants were maintained at a low aerobic exercise intensity, as defined according to the criterion of the American College of Sports Medicine, and consistent with the levels of aerobic intensity that are typically encountered during routine physical therapy after stroke. The time spent walking and resting were recorded as total therapy and total session times, respectively. Post-training activities consisted of doffing the device and recording the doff time as well as visual inspection of the paretic lower extremity for any adverse events (AEs), such as fatigue, cardiopulmonary issues (e.g., shortness of breath, dizziness), pinch points, musculoskeletal discomfort (e.g., joint pain), skin irritation, chafing, discomfort due to device slippage or weight, etc. Further, device malfunctions and clinician use errors were recorded throughout the study.

*Statistical analysis*: Longitudinal analysis included computing the differences in outcomes between baseline and post testing sessions. Gait biomechanics data from the motion capture and 10-meter timed-walk data eliminated the first and last steps from the analysis to avoid the confounding effects of gait initiation and termination. Self-reported outcomes (SIS Activity of Daily Living (ADL) and Mobility scores), only the 10-item ADL/IADL and the 9-item mobility scores were compared from pre- to post-test. All continuous data were checked for normal distribution using the Kolmogorov-Smirnov Test. If normally distributed, paired t-tests were used to compare the means at baseline and post testing, otherwise the Wilcoxon Rank Sum Test was used. SIS results pre- and post-training were compared using the Wilcoxon Rank Sum Test. The significance level was set at alpha 0.05, two-tailed. Sample size was calculated for peak swing angle and unaided gait speed data for robot assisted gait training. A total sample size of 30 was selected to allow up to 20% drop-out in the 18-training session study [9]. Performance outcomes at post- and retention timepoints were compared against the average of the two baselines.

## Results

**Fig. 6:**
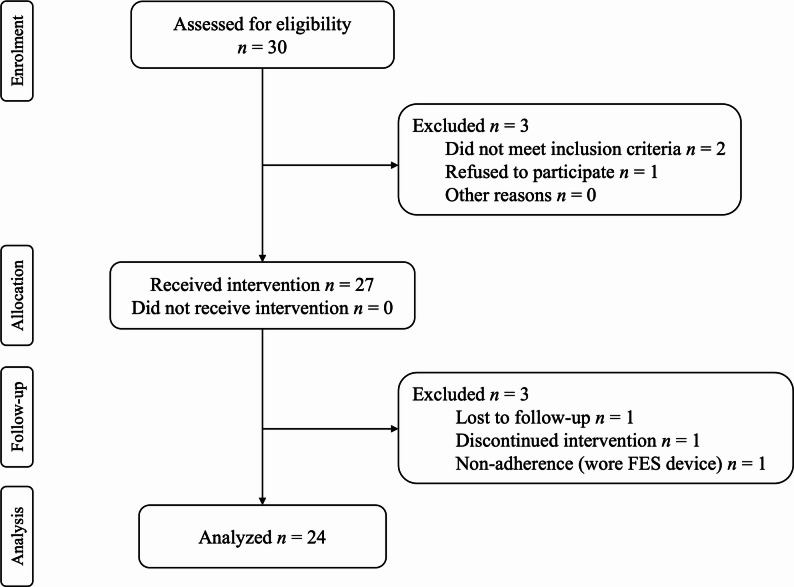
Consort Flow Chart

*Baseline Characteristics.* Baseline demographics of participants were summarized in Table 2. Thirty subjects were enrolled, with two subjects who did not meet inclusion criteria, and one who declined to participate after consent. Twenty-seven subjects started the intervention and three subjects were dropped. One subject was lost to follow-up, one was withdrawn after a motor vehicle accident, and one subject was dropped due to non-adherence (FES was worn outside the intervention). Figure 6 is the Consort Flow Chart. Twenty-four (*N* = 24) subjects completed training, including 12 male and 12 female, with a mean age of 56.5 ± 12.5 years and mean of 9.5 ± 8.5 years since stroke with hemiparesis and foot drop.

**Table 2:** Baseline characteristics of *N* = 24 subjects that completed the study

ID	Age (yr.)	TPS (mos.)	Gender	Paretic Side	AFO	AD	MMT	AROM (deg)	Speed (m/s)	Ambulation Category^*^
1	76	266	F	R	Y	Y	1+	-19	0.370	H
2	37	235	F	R	Y	N	4-	-17	0.970	C
4	70	19	M	L	N	4PC	4-	-5	0.460	LC
5	49	36	M	R	N	N	4-	-13	0.929	C
6	68	134	F	R	Y	N	4-	-8	0.550	LC
7	44	14	F	L	N	1PC	4-	-3	0.620	LC
8	34	19	F	L	Y	N	4-	-14	0.699	LC
9	58	86	M	L	N	1PC	4-	-5	0.785	LC
10	30	360	F	L	Y	N	1	0	1.000	C
11	60	233	M	L	Y	1PC	4-	-19	0.844	C
12	55	138	M	R	Y	N	4-	5	0.796	LC
14	47	56	F	L	Y	1PC	1	0	0.483	LC
16	52	95	M	L	N	1PC	2+	-15	0.603	LC
18	39	66	M	R	Y	1PC	0	-25	0.738	LC
20	65	25	F	L	Y	4PC	2-	-5	0.510	LC
21	64	308	M	L	N	1PC	4+	0	0.945	LC
22	68	17	M	L	Y	1PC	2-	-10	0.740	LC
23	58	24	M	R	N	4PC	NA	NA	0.689	LC
24	70	56	M	R	Y	4PC	1	0	0.211	H
25	69	176	F	L	Y	1PC	1+	-15	0.771	LC
26	67	210	F	L	N	4PC	1+	-20	0.291	H
27	57	100	F	L	N	1PC	3-	0	0.826	C
29	66	31	M	R	N	4PC	1	-5	0.421	LC
30	53	37	F	L	Y	4PC	1	-5	0.144	H

TPS: time post-stroke, AFO: ankle foot orthotic, AD: assistive device, MMT: manual muscle test in dorsiflexion, DGI: dynamic gait index, AROM: active range of motion in dorsiflexion, 4PC: quad cane, 1PC: single point cane. N: no cane. *Ambulation category based on walking speed: (H: home, LC: limited community, C: community ambulator) [25]

**Fig. 7:**
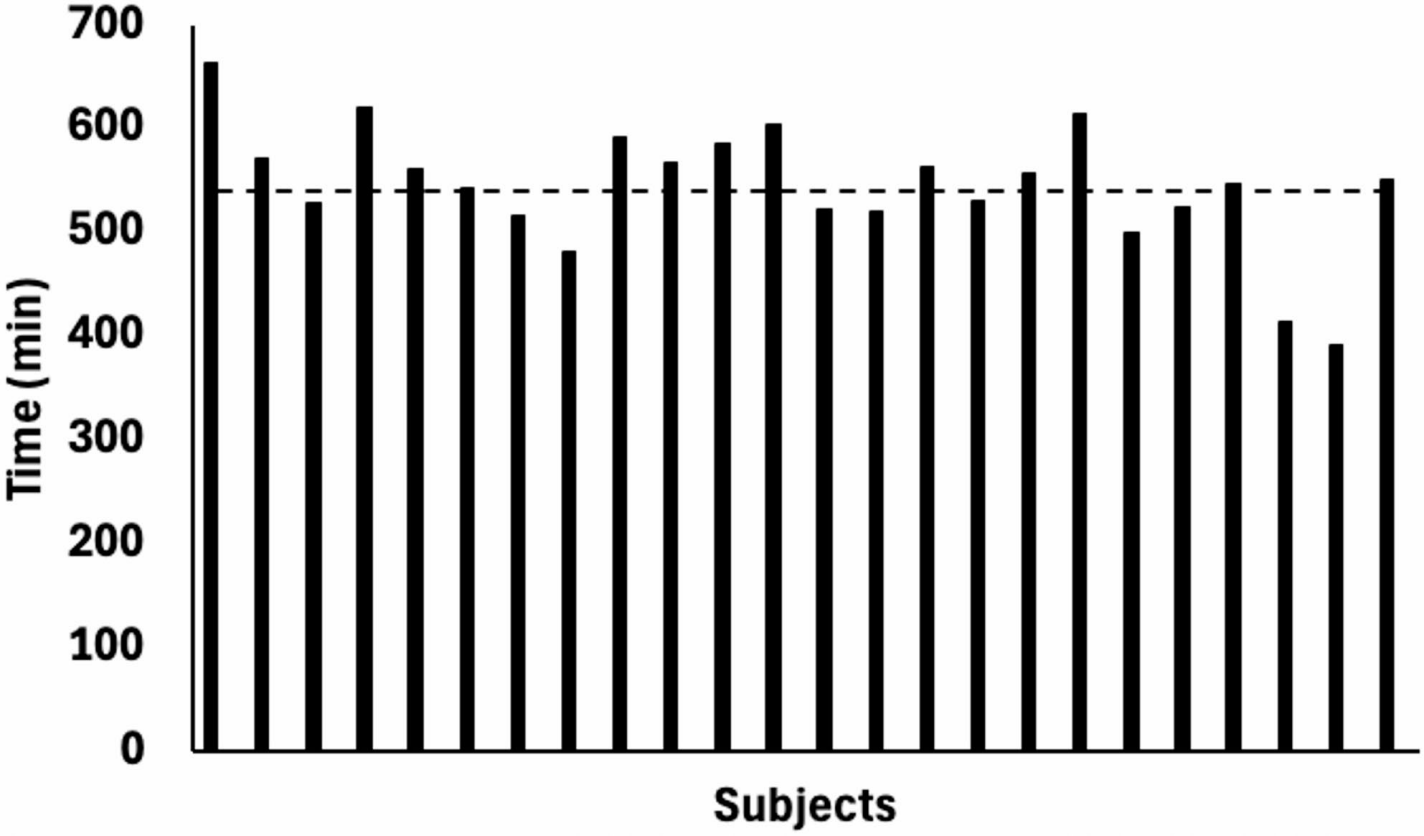
Cumulative time spent by each subject undergoing active therapy. Dashed line indicates the targeted cumulative training time goal of 540 min

*Device Usage*,* Adherence and Protocol Deviations*. Subjects were exposed to ~ 9.06 h of walking with the device with an average cumulative session duration of ~ 12 h. This reflects roughly 36 units of direct patient care (15 min per unit). On average, the group walked >1100 steps per visit, which is ~ 40% of an average person’s daily step count following stroke [24]. A majority of participants (14 out of 24 subjects) attained or exceeded the cumulative training time target (540 min) while the others tolerated between 72% and 97% of the targeted dose (Fig. 7). All participants except one completed 18 training sessions. One subject completed 17 sessions due to trainer oversight. Thus, subjects completed a total of 431 out of 432 training sessions (99.7%). Two completers were excluded from the analysis due to protocol non-adherence outside the training site—one subject wore a neuro-muscular electrical stimulation device while another wore ankle weights.

*Safety*. One subject had a serious motor vehicle accident; this was a non-study related serious adverse event (SAE) and was dropped from the study. Another subject experienced knee pain and withdrew before the intervention was started (non-device adverse event). There were a few minor device-related adverse events (AEs). One subject experienced discomfort resulting from sub-optimal fitting i.e., the smallest crampon size was not sufficiently snug and was better fit with foam pads. Two subjects had pressure and/or pinch point on shin that was resolved by adjusting the lower Velcro strap on the knee brace. One subject developed a minor abrasion on the inner thigh resulting from shoulder strap buckle, this resolved by placing foam underneath the shoulder strap buckle secured via taping. Overall, AEs were minor in severity, resolved with minor adjustments, did not result in any disruptions to the training session, and were non-recurrent. None of these events caused injuries to the trainers.

*Reliability.* Device malfunctions were reported to have occurred in < 3% of study visits. These included linear encoder errors (0.46%), premature battery discharge (0.23%), and incorrect step counts by the device (1.85%). Fuse-initiated battery trips were not considered malfunctions and instead as a safety measure by-design. Notably, there were zero Bluetooth disconnects during training. Malfunctions were resolved by restarting the device and/or the user app. In addition, there were multiple warnings related to the maximum velocity, which did not impact device safety and was addressed by implementing a more appropriate threshold for peak velocity in the device firmware. None of these device malfunctions resulted in AEs or SAEs nor did they result in the need to alter the originally planned gait training activities.

*Training Effects*. Given this was a single-arm study, participants served as their own pseudo-controls by double baseline outcome testing conducted one week apart. Functional and gait biomechanics outcomes were not statistically different between the two baseline tests except for self-selected walking speed (Table 3). The same outcome testing was performed post-training, and 2 months after all training ended. Improvements were observed in key paretic ankle-foot motor control outcomes, namely (Table 4), peak dorsiflexion angular velocity (°/s, + 12[32%], *p* < 0.01), maximum toe clearance (mm, + 10[14%], *p* < 0.01), minimum toe clearance (mm + 5[22%], *p* < 0.05), and heel-first foot strikes (%steps, + 13[40%], *p* < 0.01). Changes in peak swing angle and angle at initial contact, however, were not significant. Associated with these changes in joint kinematics, there was a significant increase in paretic step length (cm, + 3.1[8%], *p* < 0.01).

**Table 3:** Comparison of outcomes between baseline tests performed one week apart

Outcome	Baseline-1	Baseline-2	Δ
Dynamic Gait Index (0–24)	14.3	14.2	-0.1
6-Minute Walk Test (m)	249.6	254.5	4.9
10 m Self-Selected Gait Speed (m/s)	0.64	0.67	0.03^*^
10 m Fastest Gait Speed (m/s)	0.79	0.59	0.2
Peak DF Swing Angle (°)	0.5	0.2	-0.2
Angle at Initial Contact (°)	-6.6	-6.5	0.1
Maximum Toe Clearance (mm)	69.2	69.6	0.4
Minimum Toe Clearance (mm)	21.0	22.7	-0.2
Maximum DF Angular Velocity (°/s)	33.9	37.2	3.3
Knee Flexion (deg)	32.2	34.0	1.8
Hip Flexion (°)	36.1	35.9	-0.2
Hip Hike (cm)	8.8	8.7	-0.1
Hip Abduction (°)	-7.3	-8.0	-0.7
Paretic Step length (cm)	36.7	37.2	0.5
Paretic Swing (%)	38.1	38.0	-0.1
Double Support Stance (%)	26.0	25.9	-0.1
Heel-First Strikes (%steps)	31.0	31.8	0.8

^*^p < 0.05. Values reported as mean, *N* = 24

**Table 4:** Outcomes at baseline, post-test and retention endpoints (*N* = 24)

Outcome	Baseline	Post	Retention	ΔPost-Base	ΔRetention-Base	ΔRetention-Post
SIS-ADL/IADL (0-100)	72.8 ± 17.1	77.5 ± 16.8	76.8 ± 13.0	4.7	4.0	-0.7
SIS-Mobility (0-100)	80.9 ± 16.3	88.2 ± 9.5	85.6 ± 12.1	7.2 ^†,‡^	4.7	-2.5 ^*^
Dynamic Gait Index (0–24)	14.2 ± 4.6	17.3 ± 3.3	17.4 ± 3.3	3.0 ^†, ‡^	3.2 ^†, ‡^	0.1
6-Minute Walk Test (m)	252.0 ± 106.1	279.9 ± 109.1	272.8 ± 104.4	27.7 ^†^	20.8 ^†^	-7.1
10 m Self-Selected Speed (m/s)	0.66 ± 0.24	0.70 ± 0.23	0.69 ± 0.23	0.04 ^†^	0.04	-0.01
10 m Fastest Speed (m/s)	0.80 ± 0.31	0.86 ± 0.30	0.86 ± 0.31	0.06 ^†^	0.06 ^†^	0.00
Peak Swing Angle (°)	0.3 ± 6.3	0.5 ± 6.4	1.6 ± 7.1	0.3	1.3	1.1
Angle at Initial Contact (°)	-6.6 ± 4.0	-6.7 ± 4.5	-6.7 ± 5.3	-0.1	-0.1	0.0
Maximum Toe Clearance (mm)	69.4 ± 27.5	79.4 ± 30.4	78.4 ± 26.1	10.0 ^†^	9.0 ^†^	-1.0
Minimum Toe Clearance (mm)	21.8 ± 15.0	26.8 ± 20.6	23.1 ± 9.9	4.9 ^*^	1.3	-3.7
Maximum DF Angular Velocity (°/s)	35.5 ± 32.2	46.8 ± 39.7	42.5 ± 37.5	11.5 ^†^	7.0 ^†^	-4.2
Maximum PF Angular Velocity (°/s)	-101.3 ± 67.6	-111.6 ± 69.8	-100.1 ± 60.0	-10.3 ^†^	1.2	11.5
Knee Flexion (deg)	33.1 ± 16.5	35.0 ± 16.2	36.9 ± 17.7	1.9	3.9 ^*, ‡^	2.0
Hip Flexion (°)	36.8 ± 9.5	37.8 ± 8.2	39.6 ± 9.2	0.7	2.8 ^*^	1.8
Hip Hike (cm)	8.9 ± 3.2	8.8 ± 3.4	7.7 ± 4.1	-0.2	-1.2	-1.1
Hip Abduction (°)	-7.7 ± 4.8	-7.4 ± 5.4	-8.7 ± 5.7	0.3	-1.0	-1.3
Paretic Step length (cm)	36.9 ± 15.6	40.1 ± 14.2	39.1 ± 13.0	3.1 ^†^	2.2 ^*^	-1.0
Paretic Swing (%)	38.1 ± 6.6	37.1 ± 6.7	37.7 ± 6.2	-1.0	-0.4	0.6
Double Support Stance (%)	25.9 ± 6.5	25.7 ± 6.2	25.8 ± 5.5	-0.2	-0.1	0.1
Heel-First Strikes (%steps)	31.4 ± 41.3	43.9 ± 42.5	45.9 ± 42.8	12.5 ^†^	14.5 ^†^	2.0

^*^p < 0.05, ^†^*p* < 0.01, ^‡^Change greater than Minimal Clinical Important Difference. Stroke Impact Scale (SIS), Instrumental Activities of Daily Living (IADL), Dorsiflexion (DF), Plantar Flexion (PF). Values reported are mean ± SD

**Fig. 8:**
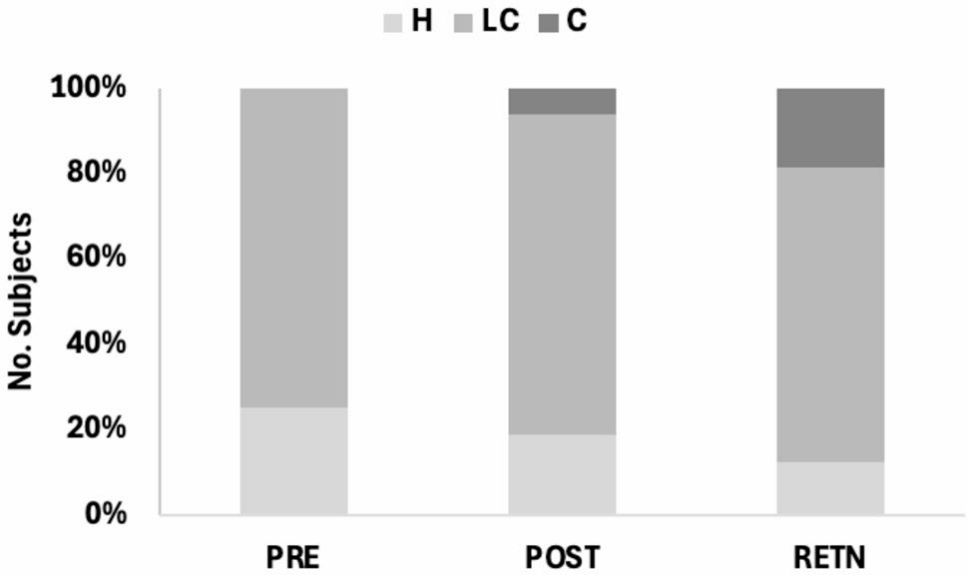
Distribution of subjects across ambulation categories [25] across testing endpoints. Eighteen subjects were home or limited community ambulators at baseline. Home (H): <0.4 m/s, Limited community (LC): 0.4–0.8 m/s, Community (C): >0.8 m/s. Ambulatory categories improve across training and after training ceases (retention [RETN], 2-month after training ends)

Participants showed statistically significant but modest improvements in their 10-meter overground gait speed, under both self-selected (m/s, + 0.04 [6%], *p* < 0.01) and fastest (m/s, + 0.06[8%], *p* < 0.01) walking conditions. Amongst participants who were either home or limited (but not full community) ambulators (*N* = 18) at baseline, one subject transitioned from a home (< 0.4 m/s) to a limited community ambulator (0.4–0.8 m/s) while another transitioned from a limited community to community (>0.8 m/s) ambulator (Fig. 8) [25]. Transitions in ambulation category continued at retention testing. Six subjects showed a small meaningful change in gait speed (>0.05 m/s, but < 0.1 m/s) while four subjects had large meaningful changes (>0.1 m/s). Clinically meaningful improvement was observed in the DGI score (+ 3-point, *p* < 0.01) but not the 6-minute timed walk distance (m, + 28[11%], *p* < 0.01). Notably, the average change in DGI was greater than the corresponding minimum clinically important difference (MCID) while the change in 6WMT trended toward its corresponding MCID i.e., 2.5 points and 34.4 m, respectively. At the individual level, 13 subjects (54%) and 9 subjects (38%) showed clinically meaningful increases in their DGI scores and 6MWT distance, respectively [25, 26].

Associated with functional improvements were significant increases in the patient-reported score on the SIS mobility subscale (+ 7[9%], *p* < 0.01), which was also greater than the corresponding MCID (4.7 points [27]), . However, the average change score on the SIS ADL/IADL subscale (+ 5[6%]) was neither significant nor clinically meaningful [27]. Within subjects, 31% reported higher than clinically meaningful changes in both ADL and Mobility scores.

*Durability of Gains Two Months after All AMBLE Training Ends (Retention).* Compared to baseline, improvements in paretic ankle-foot control outcomes (Table 4) were retained at follow up namely, peak dorsiflexion angular velocity (°/s, + 7[20%], *p* < 0.01), maximum toe clearance (mm, + 9[13%], *p* < 0.01), and heel-first foot strikes (%steps, + 13[46%], *p* < 0.01). Interestingly, significant improvements in knee flexion (°, + 4[12%], *p* < 0.05) and hip flexion (°, + 8[3%], *p* < 0.01) were evidenced at retention, but not at post-testing. Further, the change in knee flexion was greater than its MCID [28] (Table 4). Subjects also retained improvements in their 10-meter fastest (*p* < 0.01) but not their 10-meter self-selected walking speed. However, at the individual level, there were ambulation transitions between post- and retention-testing (Fig. 8), two participants transitioned from limited community (0.4–0.8 m/s) to community ambulator (>0.8 m/s) and one subject from home ambulator (< 0.4 m/s) to limited community ambulator. Further, compared to post-testing, five subjects showed a small meaningful change (0.05–0.1 m/s) while one subject had large meaningful change (>0.1 m/s).

Improvements were also retained in the DGI score (+ 3[23%], *p* < 0.01) and the 6-minute timed walk distance (m, + 21[8%], *p* < 0.01). Notably, similar to post-testing, the average change in DGI continued to be greater than its MCID. At the individual level, 15 subjects (58%) and 9 subjects (37%) showed clinically meaningful increases in their DGI scores and 6MWT distance, respectively. The average change in the SIS mobility subscale score, however, was not significant at retention testing. Within subjects however, 7 subjects (31%) demonstrated improvements greater than MCID in their SIS mobility subscale scores.

## Discussion

This study demonstrates that impedance-controlled ankle robotics, embedded in an adaptive control architecture to personalize dorsiflexion-assist during key subevents across the entire gait cycle, can durably improve gait biomechanics and functional mobility in persons with chronic HPG and foot drop, even decades after stroke. Our initial hypothesis predicted improvements in paretic ankle angle at peak swing and initial contact; but these variables did not change significantly. Instead, we found durable and clinically meaningful gains in maximal paretic toe clearance and percentage heel first strikes, key biomechanical adaptations critical for improving gait safety and efficiency. Notably, two months after all training had ended, these improvements persisted alongside the emergence of increased knee and hip flexion, marking a delayed, but important proximal lower extremity kinematic adaptation. The pertinent negative findings were that hip hike and hip abduction did not change, indicating that these pathologic compensatory gait strategies, common after hemiparetic stroke with foot drop, were not involved in the robotics mediated improvements in toe clearance and heel-first landing. Taken together, these findings showed that ankle robotics durably improves functional mobility and selected biomechanical outcomes including increasing maximal toe clearance and paretic ankle dorsiflexion angular velocity, and that 2 months after all training ended these benefits persist, along with emergence of increased knee and hip flexion as the durable adaptations improving foot drop profiles in persons with chronic HPG and foot drop after stroke.

A novel finding was that the durable effect of ankle robotics assessed 2 months after completion of training is characterized by emergence of improved knee and hip flexion. Three additional robotics-mediated biomechanical adaptations persisted durably. Two months after all training ceased, we found increased maximal toe clearance, dorsiflexion angular velocity, and percentage of heel-first strikes, these were important factors in terms of improving safety and efficiency of gait in persons with foot drop [29–32]. Contrary to our initial hypothesis, maximal toe clearance rather than ankle angle at initial contact and peak swing, emerged along with increased percent heel-first strikes as key robotics mediated features influencing the integrity of paretic foot landing. Toe clearance was a critical variable in gait safety and falls reduction post-stroke, and an outcome successfully targeted in interventions to improve safety of gait patterning for persons with HPG after stroke [29, 30]. In Parkinson’s Disease, focusing on heel strike improved toe clearance, as these two elements were critical interactive elements governing integrity of foot landing in healthy gait, as well as neurological conditions impairing gait [32–34]. Our findings that robotics durably improved gait biomechanics and safety profiles in persons up to three decades post-stroke has implications toward reducing the global burden of stroke disability, indicating a much longer therapeutic window than most stroke rehabilitation, which typically takes place in the early sub-acute period [35].

The emergence of increased paretic knee and hip flexion 2 months after robotics training ended suggests that these delayed proximal kinematic adaptations are related, at least in part, to the durably improved paretic foot landing profiles and toe clearance [31, 34, 36]. Dynamic analyses of gait illustrate that knee and hip flexion and their timing are critical to the multi-segmental motor control of limb shortening for foot clearance, and that these factors synergistically modulate foot landing in healthy and neurological gait [34, 36, 37]. We do not know why the increased knee and hip flexion emerged as significant kinematic improvements 2 months after robotics training ended. Notably, this unexpected finding is delayed in relation to the temporal proximity of robotics training. This raises the possibility that a robotics related sensorimotor learning pattern, or proximal after-effect improved toe clearance and foot landing without knee and hip flexion having changed, but that this pattern changes after 2 months to be replaced by multi-segmental (knee and hip flexion) kinematic synergies that evolve when persons with HPG walk on their own for several months after all robotics training has ended [10, 11, 38, 39].

*Relevance of improved joint kinematics*: Tripping over an obstacle is one of the biggest causes of falling in the elderly [40, 41]. AMBLE participants improved their ankle sensory motor control, as demonstrated by greater toe clearance, sufficient to clear typical obstacles encountered at home ranging from < 1 cm to 8 cm [42]. Improvements in ankle control were also evidenced by a higher incidence of heel-first foot strikes. Digitigrade walking, as observed and manifesting as toe-drag in persons with foot drop, increases the external mechanical work performed by the limbs. In particular, this requires a higher energy cost of transport [43] and imposes an excessive kinetic burden that increases the risk of knee injury, including but not limited to anterior cruciate ligament injuries [44]. Alternately, lateral foot contact is also unsafe as it can predispose to ankle sprains, especially given that chronic stroke patients often have weak ankle evertor musculature, and increased tone resulting in ankle inversion. In contrast, walking with a heel-first strike pattern can reduce the loading forces of the knee joint [44] and has been shown to improve the economy of walking [43]. Participants also experienced significant increases in their peak DF velocity, which has been shown to be a valid and reliable task-specific measure of ankle dorsiflexion during walking [45]. Collectively, these durable robotics mediated improvements in toe clearance, foot landing, and ankle dorsiflexion velocity have implications for reducing falls after stroke, which population-based studies show occur in about 37% of six-month survivors, along with high injury rates requiring medical treatment [46].

*Improvements in walking function*: Slower speed has been associated with increased risk of falls, even after adjustments for potential confounders and traditional clinical tests of cognition, gait, and balance [47]. In fact, each 10 cm/s decrease in gait speed has been associated with a 7% increased risk for falls and community-dwelling older adults with slow gait speed (≤ 0.7 m/s) demonstrate a 1.5-fold increased risk for falls compared with those with normal speed [40]. While the average increase in gait speed was modest, the group trended towards a higher ambulation category from limited community ambulator (0.4–0.8 m/s) to community ambulator (>0.8 m/s) [48]. Within the group, the largest transition was from limited to full community ambulators (12%). Further, a majority (68%) presented small meaningful changes (>0.05 m/s) while a significant proportion (38%) demonstrated large meaningful changes (>0.1 m/s) in walking speed. Participants also improved their dynamic balance trending toward safe ambulators (DGI score) as well as endurance (6MWT distance), with changes in both being higher than their corresponding minimum clinically important differences (MCID’s) [25, 26]. The clinical meaningful change for DGI is + 3 points. For the DGI, the 15 individual subjects had a greater 3-point change in DGI values at both post and retention timepoints, but there were 13 subjects that retained the same level of clinically meaningful change at the post and retention timepoints. The clinical meaningful change for 6MWT is + 34.4 m. For the 6MWT, the 9 individual subjects had a greater than MCID change in 6MWT values at both post and retention timepoints, but there were 7 subjects that retained the same level of clinically meaningful change at the post and retention timepoints. These improvements reflect faster and, more importantly, safer walking over longer distances, which is also corroborated by more than 30% of subjects self-reporting clinically significant improvements in mobility function (SIS Mobility score). However, the magnitude of the improvements in 10-meter walking and 6-minute distance are not robust; they are modest. This may be due in part to the chronicity of our stroke study population, as time after stroke is reported to be an important co-variate known to attenuate treatment effects on walking outcomes in both exercise and robotics gait training interventions [49, 50]. Our findings may also be due to the nature of the training stimulus, with capitation of walking intensity at low to moderate aerobic intensity, as higher intensities are known to be more effective for locomotor recovery [51]. Regardless, our findings suggest that the adaptive response to ankle robotics training in chronic HPG is more robust and durable for biomechanical improvements. We posit that precisely timed, assistance as-needed technology enhanced voluntary ankle-foot control, resulting in sustained biomechanical improvements in toe clearance and heel first strikes during unassisted walking. The persistence of these improvements at the 2-month follow-up represents a novel finding, indicating successful sensorimotor learning of discrete sub-tasks during the gait cycle through targeted robotic intervention.

*Comparisons to usual care*: We compared our findings to studies utilizing usual treatment physical therapy (conventional gait training) for chronic patients (>6 months since stroke). A meta-analysis of 9 studies involving 499 participants highlighted “little benefit in training chronic stroke in overground walking,” except modest gains in timed walks [52]. Specifically, based on 3 studies with 269 participants, it found “no evidence for a benefit on the primary variable, post-test gait function,” as assessed using multi-dimensional, ordinal scales that are validated for use in stroke. Increases in 10-meter gait speed (0.07 m/s) and six-minute walk distance (26 m) were reported based on 7 studies with 396 participants and 4 studies with 181 participants, respectively. A summative finding was that “insufficient evidence [exists] to determine if overground physical therapy gait training benefits gait function in patients with chronic stroke, though limited evidence suggests small benefits for unidimensional variables such as gait speed or 6MWT.”

In contrast, AMBLE robotic therapy increased gait speed by 0.04 m/s (comfortable) and 6WMT distance by ~ 21 m, which is lower than but comparable to, those resulting from usual care [52]. Lower changes in gait speed outcome measures may be attributable to the training objective itself: training was conducted at participant’s self-selected walking speeds. Unlike high-intensity, treadmill walking exercise protocols that progress walking speed, this study emphasized voluntary ankle sensorimotor locomotor learning rather than gait speed. Indeed, as hypothesized, significant improvements were evidenced in key gait biomechanical outcomes. Improvement in dynamic balance (DGI score) may arise as a result of improved ankle sensory motor learning and changes up the kinematic chain at the knee and hip joint. We compared our results against two studies employing usual care interventions [53, 54]. In comparison to other gait training approaches, including rhythmic auditory cueing [53] and partial weight suspension treadmill training [54], DGI scores reported here were significantly higher, had larger effect size to reach meaningful clinical significance and were durable. AMBLE participants were also more chronic (9.5 years) than those reported in studies elsewhere [52] (< 7 years). A more detailed comparison is precluded, however, as this study reports changes in biomechanical surrogates that are relevant to foot drop (heel-first strikes, dorsiflexion angular velocity, toe clearance) that to our knowledge are not reported elsewhere [55]. Thus, we posit that the durable biomechanical and functional gait changes as evidenced in our study would not be expected from gait training alone in chronic stroke.

*Study limitations*: This study is limited by the small sample size, non-controlled design, and restriction to chronic stroke. The non-controlled study design in this Phase 2 study for persons with chronic stroke was approved by our National Institute of Health – National Institute of Neurological Disorders and Stroke (NINDS) cooperative study advisors following Phase I findings that two weeks training (3xweekly) using only the weight of the robot (AMBLE worn in the off-state; no actuation) produces no significant biomechanical or functional gait changes in chronic hemiparetic stroke participants. Stability across repeated baseline testing is interpreted in the current report as evidence that the current locomotor learning is mediated by task-specific training with the actuated AMBLE in an otherwise stable neurological cohort. Randomized clinical trials with a reference-treatment control group in persons with sub-acute stroke are ongoing to investigate whether AMBLE improves hemiparetic gait during the earlier sub-acute stroke recovery. Notably, the small sample size and study entry restriction to persons with only mild to moderate severity HPG prevents subset analysis of how different deficit severity profiles might differentially respond to robotics treatment. This study cannot address the question, as to whether robotics interventions initiated early may elicit greater adaptive neuroplasticity response, as our participants are a mean of a decade post stroke.

A key limitation is that we did not measure and do not know the mechanisms underlying the efficacy of robotics to improve gait biomechanics after stroke. Based on engineering models and prior studies, we posit that impedance control may optimize volitional human-robotic cooperative learning [49, 50]. We posit that robotics assisted gait training mediated neuroplasticity alters midbrain-cerebellar networks, as shown with treadmill training to improve gait in chronic stroke and recognized as the key neuroanatomic locus for bipedal gait in sub-human primates, learned locomotor behavior in cats, and neural control in human gait and postural control [56–60]. Regardless, our findings suggest that robotics training exposure of ~ 9 h divided across 18 session mediates functional neuroplasticity to improve gait biomechanics, in this study, one to 30 years after the stroke event. Further development of robotics control systems targeted to mediate functional neuroplasticity are needed to better understand the mechanisms and their potential to reduce the disability of neurological disease.

## Conclusions

Ankle robotics training using AMBLE durably improves critical gait biomechanics, including toe clearance, heel-first strikes, and ankle dorsiflexion velocity, in individuals with chronic HPG. The delayed emergences of increased knee and hip flexion highlight the potential for robotics to induce long-term kinematic adaptations along the lower limb chain. These findings support the role of robotics-assisted rehabilitation as a transformative approach for addressing chronic gait deficits after stroke. Larger randomized clinical trials are needed to determine the effectiveness of robotics assisted rehabilitation across different and earlier phases of stroke recovery, and to critically evaluate the added benefits of robotics integrated stroke rehabilitation compared to best conventional care.

## Data Availability

The datasets associated with the study are not publicly available due to patient data protection policy but are available from the corresponding author on reasonable request. Information on this clinical trial (Clinical Trial Identifier: NCT04594837) can be found at: [https://clinicaltrials.gov/study/NCT04594837](https://clinicaltrials.gov/study/NCT04594837) .

## Abbreviations

1PC: Single point cane
3-D: Three dimensional
4PC: Quad cane
6MWT: Six-minute walk test
AAN: Active assist as-needed
AD: Assistive device
ADL: Activity of Daily Living
AEs: Adverse events
AES-128-CTR: Advanced Encryption Standard 128 bit Counter
AFO: Ankle foot orthosis
AMBLE: Assisted Mobility and Balance for the Lower Extremity
AROM: Active range of motion
C: Community
cm: Centimeter
deg: Degree
DF: Dorsiflexion
DOF: Degree of freedom
DGI: Dynamic Gait Index
e.g.: For example
EHO: Electro-hydraulic Ankle-Foot Orthosis
F: Female
FES: Functional electrical stimulation
g: Gram
H: Home
h: Height
HPG: Hemiparetic gait
IADL/ADL: Instrumented/activities of daily life
kg: Kilogram
L: Left
LC: Limited community
M: Male
mAh: Milliampere hour
MCID: Minimum clinical significant difference
m: Meter
min: Minute
mm: Millimeter
N: No
N: Number
Ni: Nickel
NINDS: National Institute of Neurological Disorders and Stroke
NP: Non-paretic
NYHA: New York Heart Association
P: Paretic
PT: Physical therapy
PWM: Pulse width modulator
R: Right
RETN: Retention
ROM: Range of motion
s: Second
SAE: Serious adverse events
SD: Standard deviation
SST: Single support stance time
SIC: Short interval control
SL: Step length
SIS: Stroke Impact Scale
t: Time
t_h_: Time hold
t_o_: Toe off
t_rise_: Rise time
vs: Versus
Y: Yes
yrs: Years
